# Association between acoustic speech features and non-severe levels of anxiety and depression symptoms across lifespan

**DOI:** 10.1371/journal.pone.0248842

**Published:** 2021-04-08

**Authors:** Luciana Albuquerque, Ana Rita S. Valente, António Teixeira, Daniela Figueiredo, Pedro Sa-Couto, Catarina Oliveira

**Affiliations:** 1 Institute of Electronics and Informatics Engineering of Aveiro, University of Aveiro, Aveiro, Portugal; 2 Center of Health Technology and Services Research, University of Aveiro, Aveiro, Portugal; 3 Department of Electronics Telecommunications and Informatics, University of Aveiro, Aveiro, Portugal; 4 Department of Education and Psychology, University of Aveiro, Aveiro, Portugal; 5 School of Health Science, University of Aveiro, Aveiro, Portugal; 6 Center for Research and Development in Mathematics and Applications, University of Aveiro, Aveiro, Portugal; 7 Department of Mathematics, University of Aveiro, Aveiro, Portugal; VU medisch centrum School of Medical Sciences, NETHERLANDS

## Abstract

**Background:**

Several studies have investigated the acoustic effects of diagnosed anxiety and depression. Anxiety and depression are not characteristics of the typical aging process, but minimal or mild symptoms can appear and evolve with age. However, the knowledge about the association between speech and anxiety or depression is scarce for minimal/mild symptoms, typical of healthy aging. As longevity and aging are still a new phenomenon worldwide, posing also several clinical challenges, it is important to improve our understanding of non-severe mood symptoms’ impact on acoustic features across lifetime. The purpose of this study was to determine if variations in acoustic measures of voice are associated with non-severe anxiety or depression symptoms in adult population across lifetime.

**Methods:**

Two different speech tasks (reading vowels in disyllabic words and describing a picture) were produced by 112 individuals aged 35-97. To assess anxiety and depression symptoms, the Hospital Anxiety Depression Scale (HADS) was used. The association between the segmental and suprasegmental acoustic parameters and HADS scores were analyzed using the linear multiple regression technique.

**Results:**

The number of participants with presence of anxiety or depression symptoms is low (>7: 26.8% and 10.7%, respectively) and non-severe (HADS-A: 5.4 ± 2.9 and HADS-D: 4.2 ± 2.7, respectively). Adults with higher anxiety symptoms did not present significant relationships associated with the acoustic parameters studied. Adults with increased depressive symptoms presented higher vowel duration, longer total pause duration and short total speech duration. Finally, age presented a positive and significant effect only for depressive symptoms, showing that older participants tend to have more depressive symptoms.

**Conclusions:**

Non-severe depression symptoms can be related to some acoustic parameters and age. Depression symptoms can be explained by acoustic parameters even among individuals without severe symptom levels.

## Introduction

The World Health Organization (WHO) recognizes that psychological disorders, such as depression and anxiety, are major public health concerns defined by a combination of atypical perceptions, thoughts, behaviors, emotions and relationships with others [1]. Depression is the world’s fourth most significant etiology of disability, leading to high costs for governments worldwide [2]. Psychological conditions present a global impact on individuals and on their quality of life [3].

The diagnostic process of depression and anxiety is based on assessment tools that rely on the patients’ perception of their symptoms and/or on the clinicians’ opinion about the interview style [3]. Consequently, the diagnostic process is subjective and time-consuming, requiring training and practice to produce a reliable result [4]. Measurable biomarkers, such as speech, could contribute and assist specialists in a more accurate and objective detection of symptoms and, consequently, in the selection of a more effective treatment [5, 6]. Due to its highly complex production, speech has shown to change along with the cognitive and physiological changes that result from mental health symptoms [7, 8]. Speech can be studied in their fully extension, comprising both segmental and suprasegmental features. Segmental features concern the characteristics of individual phonemes; suprasegmental or prosodic features are transmitted in syllables, utterances, or sentences and consists in, e.g., acoustic emphasis, rhythm, stress or intonation [9].

The analysis of the influence of diagnosed anxiety/depression disorders in acoustic parameters allows the collection of information that can contribute to the development of automatic detection systems of mood disorders to support the diagnosis based on measurable biomarkers (behavioral, biological and physiological features). Also important, but less studied, is how acoustic features are associated with minimal (i.e., subclinical) mood symptoms.

Research studies that focus on that subject will contribute to an early and more reliable recognition of mood disorders. Additionally, speech and language pathologists, the professionals responsible for the intervention on voice alterations, could increase their knowledge of the variation of acoustic features in people with anxiety and/or depression symptoms, contributing to the differentiation between alterations derived from voice disorders and variations derived from minimal-to-mild mood symptoms.

### Anxiety symptoms and acoustic features

Fear, tension and distress are common symptoms associated with anxiety, usually assessed by subjective methods [10, 11]. As anxiety disorders have a reflection in people’s voice due to the somatic symptoms associated with the respiratory system, the acoustic parameters could be used as an objective method to assist in the assessment of anxiety symptoms [11, 12]. Several research studies had evidenced the influence of anxiety symptoms in acoustic parameters. According to Banse and Scherer [13], Hagenaars and Minnen [14], Diamond et al. [15], Weeks et al. [16], Low et al. [3], the mean fundamental frequency (F0) increase in individuals with anxiety. The variability of F0 was also evidenced to be a good indicator of anxiety symptoms, according to Hagenaars and Minnen [14] and Goberman et al. [17], reporting a higher pitch variability with the increase of anxiety. Other researchers, in contrast, found different trends in this acoustic variable [18–20].

Suprasegmental measures, such as percent pause time and number of pauses were proven to positively correlate with the increase of anxiety [10, 17, 21]. Although, speech rate tends to increase with anxiety increase [10, 14, 22].

Increased anxiety also leads to higher jitter and shimmer values [3, 23]. Loudness and harmonic-to-noise ratio (HNR), on the other hand, have an irregular performance, presenting distinct results in different research studies—either no change, decrease or increase [21, 22, 24].

Ozseven et al. [11] analyzed a broader set of acoustic parameters (122 acoustic measures) in patients diagnosed with anxiety and in healthy individuals and observed that 42 acoustic parameters (e.g., F0, F1, jitter, shimmer, mel-frequency cepstrum coefficients (MFCCs), and wavelet coefficient) change, in different directions and intensities, with anxiety. For example, F0 mean, F1 mean, jitter, shimmer and wavelet coefficients increase in anxious patients and, in general, MFCCs decrease with anxiety.

### Depression symptoms and acoustic features

Depression cause changes in the somatic and automatic nervous system that reflects on muscle tension and respiratory rate [25, 26]. Those changes have an impact on prosody and speech quality [27–29]. The increase muscle tension and changes in salivation and mucus secretion affects vocal tract and limits articulatory movements, leading to articulation errors, reduce pitch range, decrease in speech rate and increase hesitations [25, 30]. In a vast amount of research studies, the reduction of F0 range and the F0 average are found to be linked with depression severity [3, 4, 31–33]. F0 range was also evidenced to be a biomarker in treatment responders, as pitch variability increase significantly in patients that present depressive symptoms decrease [4, 34].

The slowing of thoughts and reduction of physical movements that occur in depression—psychomotor retardation (PMR)—could explain the reduction in F0 parameters, as the complexity of the larynx neuromuscular system is affected by disturbances in muscle tension due to PMR [30, 35–38]. The increase of muscle tension in the vocal tract could also explain the tightening of the vocal folds and, consequently, less variable speech [25, 30, 38–41]. Although, other studies did not find a significant correlation between the F0 parameters of depressed and non-depressed patients, possibly due to methodological aspects or the intrinsic characteristics of F0 (i.e., simultaneously an indicator of the affective status and a marker of the physical state of vocal folds) [4, 30, 41].

Similarly to F0, contradictory results concerning variation in loudness were found in the literature, whereas only some research studies showed statistically significant improvements of energy parameters after depression treatment [27, 33].

More consistent results were found related to the other prosodic feature: speech rate. Cannizzaro et al. [30] found evidence of a strong negative correlation between speech rate and a clinical subjective rating of depression. Investigations using different sample sizes conclude, in general, that speech rate is reduced in individuals with depression [4, 30, 42–44]. A study [45], considering phonologically-based measures of speech rate, observed stronger correlations of these measures with depression status and subjective measures of depression, when compared with a global speech rate value. Despite the value of speech rate as a potential biomarker of depression severity, it remains unclear whether the reduction in speech rate is an indicator of motor retardation or lower cognitive functioning [5, 27, 30, 45]; additionally, speech rate could not present appropriate discriminatory evidence to be a single biomarker of depression [5].

Formant measures represent acoustic resonances of the vocal tract. Considering that depression could affect vocal tract properties, formant features are also suitable as a marker of these changes [4, 5, 46]. Several studies have [4, 46–49] found a decrease in formant frequencies in comparison with healthy individuals. This finding could be explained by PMR that causes either tightening on vocal tract or lack of motor coordination [41, 45–47, 50].

Further voice measures, such as jitter, shimmer and HNR are voice quality measures that are positively correlated with depression [3, 41, 49, 51]. Indirectly-relevant features of voice properties (e.g., MFCCs or power spectral density) are also correlated with individuals’ mood [6, 47, 50–52]. Taguchi et al. [6] investigated the differences in the MFCCs on individuals with and without depression and found evidence of higher levels of sensitivity and specificity of the second dimension of a MFCCs, concluding that this dimension could be a discriminatory factor between depressed and healthy patients and, consequently, a depression biomarker.

Suprasegmental speech measures were also found to have significant correlation with subjective measures of depression [4, 30]. Total recording duration increased with depression severity due to more variable and longer pauses, which resulted in a decrease in speech to pause ratio [4, 27]. Percent of pause time is higher in the depressed group [30]. The studies of Mundt et al. [4] and Mundt et al. [34] also revealed that total recording duration, total pause time and number of pauses showed a significant decrease in patients that respond positively to depression treatment, so these measures could be considered as biomarkers to monitor treatment progress. By contrast, patients that do not respond to treatment presented smaller vocal acoustic changes or even no changes.

### Objective

The acknowledgment that different acoustic features could be associated with depressive and/or anxiety symptoms lead to the exploration of this relationship in a sample composed by adult participants of different ages. Therefore, the present study intends to 1) analyze the association between the acoustic parameters of vowels in stress position with depressive and anxiety symptoms 2) analyze the association between suprasegmental characteristics of spontaneous speech (e.g., rhythmic measures, speaking F0 and HNR) with anxiety and depressive symptoms. So, the aim of this study is to determine if variations in segmental and suprasegmental acoustic features have corresponding alterations in anxiety or depression symptoms in adult population across lifetime.

## Method

All ethical procedures were ensured prior to any data collection for this cross-sectional study. The project was submitted and approved by the Ethics Committee Centro Hospitalar São João/ Faculty of Medicine, University of Porto, Portugal (number N38/18). All participants agreed and signed the written consent form before participating in the study.

### Participants

A convenience sample of 112 adult Portuguese speakers (aged between 35-97) participated in this study, and were divided into 4 age groups [35-49] (15 men, 15 women), [50-64] (15 men, 15 women), [65-79] (15 men, 16 women), and ≥80 (11 men, 10 women). To be included, participants had to meet the following inclusion criteria: be Portuguese native speaker; no history of speech-language impairment, severe hearing problems, neurological conditions or head/neck cancer; be able to follow instructions; absence of upper respiratory tract infection for 3 weeks before the speech collection; absence of currently smoking habits or in the previous 5 years; good general health reported by self-assessment; absence of hearing aids.

The data used in the current study were originally collected in a large ongoing project concerning the analysis of the effects of age and gender on acoustic variables (i.e., F0, F1, F2 and duration of European Portuguese language (EP) oral vowels) [53] and suprasegmental measures derived from spontaneous speech. For more details see Albuquerque et al. [53].

The participants also fulfilled a questionnaire and an instrument concerning anxiety and/or depressive symptomatology (described below), whose data was studied in the present research.

### Instruments

Each participant completed a background questionnaire, which intends to collect information concerning age, gender, educational level and habits. The Hospital Anxiety Depression Scale (HADS), a self-report questionnaire, was used to evaluate anxiety and depression symptoms. HADS is not a time-consuming instrument, and has been largely used in research studies and in clinical settings with non-psychiatric populations [54]. It presents good internal consistency, sensitivity and specificity and concurrent validity with questionnaires commonly used to assess anxiety and depression [55]. HADS is divided into an Anxiety subscale (HADS-A) and a Depression subscale (HADS-D) with seven items each. Each item has a 4-point Likert score scale with a minimum value of 0 and a maximum value of 3. Higher scores represent higher levels of anxiety and depressive symptoms. The HADS manual provides cut-offs scores indicating mild (8–10), moderate (11–14), or severe (15–21) anxiety or depression [54, 56, 57]. Following the cut-offs, a score of 0–7 for each subscale could be regarded as being without anxiety or depression symptoms [54]. So, 7 is the maximum value for the normal range.

### Corpus and recording protocol

The corpus consists of two types of parameters: the first refers to segmental and the second to supragmental acoustic measures.

#### Segmental

The speech corpus for segmental analysis consisted of 28 disyllabic words, with the EP vowels [i], [e], [ε], [a], [o], [ɔ] and [u] in stressed position, mostly composed by a C**V**.CV sequence.

The consonants used in the sequence were voiced/voiceless stop consonants or voiced/voiceless fricatives. The stimuli were embedded in a carrier sentence “Diga…por favor” (“Say…please”). Four different words were selected for each vowel. The words were chosen based on familiarity and easiness of graphical representation to overcome interferences of reading difficulties [58].

The randomized sentences were presented individually on the computer screen using the software system SpeechRecorder [59], where picture and orthographic words could be viewed simultaneously. After the participant became acquainted with the sentences structure, the researcher asked the participant to read the sentence at a comfortable loudness and pitch level. Each sentence was repeated three times, in a total of 12 repetitions of each vowel, 84 productions by participant (112 participants x 28 words x 3 repetitions = 9408 recordings).

#### Suprasegmental data

The participants were also instructed to describe the standardized picture “Cookie Theft picture” [60] in order to analyze spontaneous speech.

All recordings took place in quiet rooms, in which participants were seated at a table and their speech productions were recorded using an AKG C535 EB cardioid condenser microphone connected to an external 16-bit sound system (PreSonus Audio-BoxTM USB) in a sampling rate of 44100 Hz.

### Segmentation

Concerning data obtained from the production of disyllabic words, WebMAUS general [61, 62] was used to automatically segment the recordings at word and phoneme level. Data was then imported into Praat speech analysis software [63] and manually analyzed by four trained raters who checked the accuracy of vowel boundaries. Data with clipping, recording artifacts (e.g., noise or cough), with unusual hoarseness/ vocal fry or misread words were excluded from the analysis in a total of 6% of the total data [64].

Related to spontaneous speech, a Praat script [65] was used to automatically detect silent pauses of over length 250 ms [30] and create textgrid files. The automated alignments were manually checked by two trained analyzers, who verified the accuracy of pause and speech intervals. Speech intervals with speaker and/or environmental noise were not considered for further analysis, and also the beginning and end of all recordings were not considered in the analysis due to sentence initial and final acoustic variability (a total of 7% of the speech intervals were excluded).

### Acoustic features

A set of 18 parameters were extracted from the recording data. As the recordings were not conducted with this primary aim, it was not possible to measure all voice cues that are susceptible to change due to mood symptoms. The chosen parameters represent the acoustic features mostly used in this research field and also those that reflect alterations in the dynamics of speech production with a change in motor control related to depressed and/or anxiety symptoms [10, 39]. Parameters are defined in Table 1. The following procedures were adopted in the extraction of data for the segmental and suprasegmental domains.

**Table 1:** Description of the segmental and suprasegmental parameters used.

**(a) Segmental parameters**	**Description**
Vowel Fundamental Frequency (Hz)	Median of vibrations per second of the vocal folds on vowels (F0)
Vowel Formant Frequencies (Hz)	The resonance frequencies of the vocal tract (F1 and F2) on vowels
Vowel duration (s)	Mean duration of all EP stressed oral vowels
**(b) Suprasegmental parameters**	**Description**
Total speech duration (s)	Sum of speech duration of all speech intervals
Total pause duration (s)	Sum of pause duration of all pause intervals
Total recording duration (s)	Sum of all speech and pause intervals
Percent pause time (%)	Total pause duration divided by total time (all speech and pause intervals)
Speech pause ratio	Total time talking divided by total pause time
Number of pauses	Number of pause intervals in the description task
Mean pause duration (s)	Duration average of pause length
Mean speech duration (s)	Duration average of speech length
Pause variability (s)	Standard deviation (SD) of pause length
Speech variability (s)	Standard deviation (SD) of speech length
Number of syllables	Sum of all syllable onset detected within all speech intervals
Speech rate (syllables/s)	Number of syllables divided by the total time (include pause intervals)
Speaking F0 (Hz)	Average number of vibrations per second of the vocal folds in the entire speech sample
HNR (dB)	Average ratio of the aperiodic energy to the harmonic energy

#### Segmental data

F0, formant frequencies (F1 and F2) and vowel duration were automatically extracted from segmented data using Praat scripts. The cross-correlation algorithm was used to estimate F0 of the vowels, which is suitable for short vowels [66]. F0 median value was obtained from the central 40% of each target vowel, thus minimizing the impact of flanking consonants on F0. The median value was obtained instead of mean F0 to decrease the impact of F0 measurement errors [66]. The pitch range used for F0 analysis was 60-400 Hz for male and 120-400 Hz for female. The burg-LPC algorithm provided by Praat was used to compile values for F1 and F2 at the central 40% of the vowel. A procedure adapted from [66] and previously used in Albuquerque et al. [67] and Oliveira et al. [68] was applied to optimize the formant ceiling for a certain vowel of a certain speaker. F1 and F2 were calculated 201 times for each vowel, for all ceilings between 4500 and 6500 Hz in 10 Hz steps (for female) and between 4000 and 6000 Hz in 10 Hz steps (for male). The ceiling referred above was chosen as the one that produced the lowest variation.

Vowel duration was obtained from the annotation files considering the beginning and ending points of each vowel and vowels shorter than 20 ms were excluded.

#### Suprasegmental data

For syllable count, an adapted Praat script of the BeatExtractor [69, 70] was used to detect vowel onset using a beat wave (a normalized and band-specific amplitude). The cut-off frequency were defined automatically, the thresholds were 0.1 (threshold 1) and 0.06 (threshold 2), the filter was defined as Butterworth and the technique was Amplitude.

To obtain speaking F0 automatically from the description picture task a Praat script (Prosody Descriptor) [71] was used to measure mean F0 in valid speech intervals, with the threshold 75-400 Hz for males and 120-600 Hz for females. Each value was considered and used to obtain the average of speaking F0 for each participant.

### Statistical analysis

All acoustic and mood data were compiled in a SPSS file (IBM SPSS software package version 25.0; SPSS Inc., Chicago, IL, USA) [72]. The segmental measures (F0, F1, F2 and duration) were obtained for each vowel and, afterwards, median of repetitions was obtained for each vowel type and speaker. F0, F1, F2 and duration mean for stressed vowels were also calculated. The suprasegmental measures (presented in Table 1) were also incorporated.

Descriptive data for HADS-A and HADS-D were obtained through the calculation of mean and standard deviation by age (both in a categorical and continuous format), and gender. A two-way ANOVA was performed, including the interaction term between age group and gender. The variance homogeneity (Levene test) and the normality of residuals (by using inspection of QQ plot) were verified. Additionally, descriptive data for segmental and suprasegmental acoustic parameters were reported in mean and standard deviation by gender and HADS-A or HADS-D mood symptoms classification (≤7 versus >7, respectively). Adopting the intensity of change used by [11], in the comparison of neutral reading and anxious reading/spontaneous speech, which considers that a high increase is superior to 10% and a high decrease exceeds -10%, the differences between speakers without anxiety/depression symptoms and speakers with mood symptomatology were analyzed by gender.

To explore and model the relationship between all acoustic variables and the scores of mood symptoms (either HADS-A or HADS-D), a multiple linear regression model was developed with non-highly correlated acoustic variables as independent variables (defined as multivariable model). Then the regression models were adjusted for age (continuous) and gender (defined as adjusted model). The assumptions of residuals normality (QQ plot inspection) and homoscedasticity (scatterplot of residuals versus predicted values) were verified. The multicollinearity between independent variables were evaluated by Pearson correlation. Correlation values superior than 0.70 (in module) were considered highly correlated. Acoustic variables that presented a very large (> 0.7) magnitude of correlation [73], meaning that they measure the same behaviour and present a similar contribution to the model [74], were excluded from the analysis. So, only the acoustic variables vowels F0, vowel duration, vowels F2, total speech duration, total pause duration, speech rate, percent pause time and HNR are included. Due to multiple testing, resulting from the regression models (four models at total), the significant level used was 0.0125.

## Results

First, this section presents the sample characterization in terms of HADS-A and HADS-D scores by gender and age group. Secondly, the association of HADS-A and HADS-D with acoustic parameters are presented.

### Sample characterization concerning mood measures

**Fig 1:**
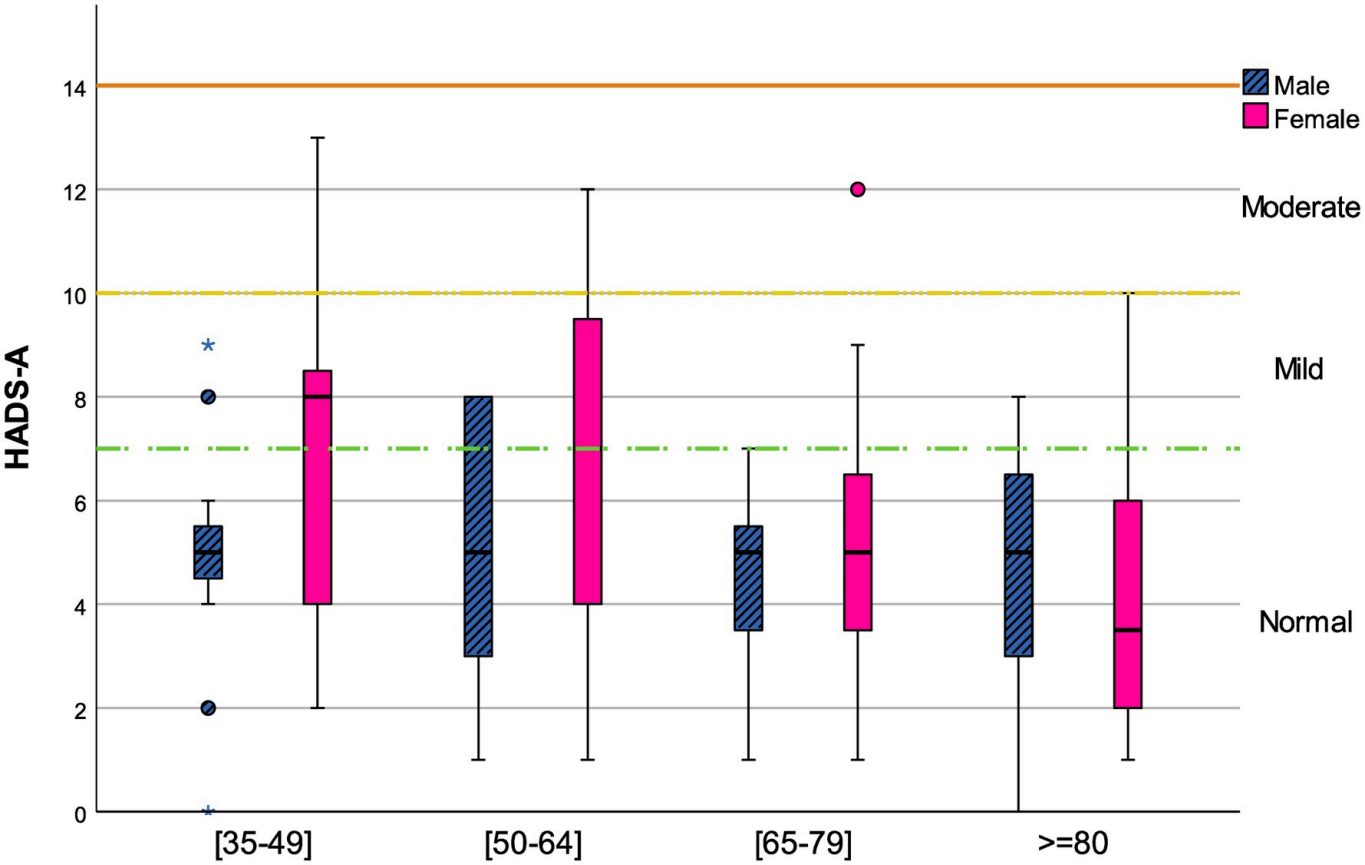
Age effect on HADS-A for both genders.

**Fig 2:**
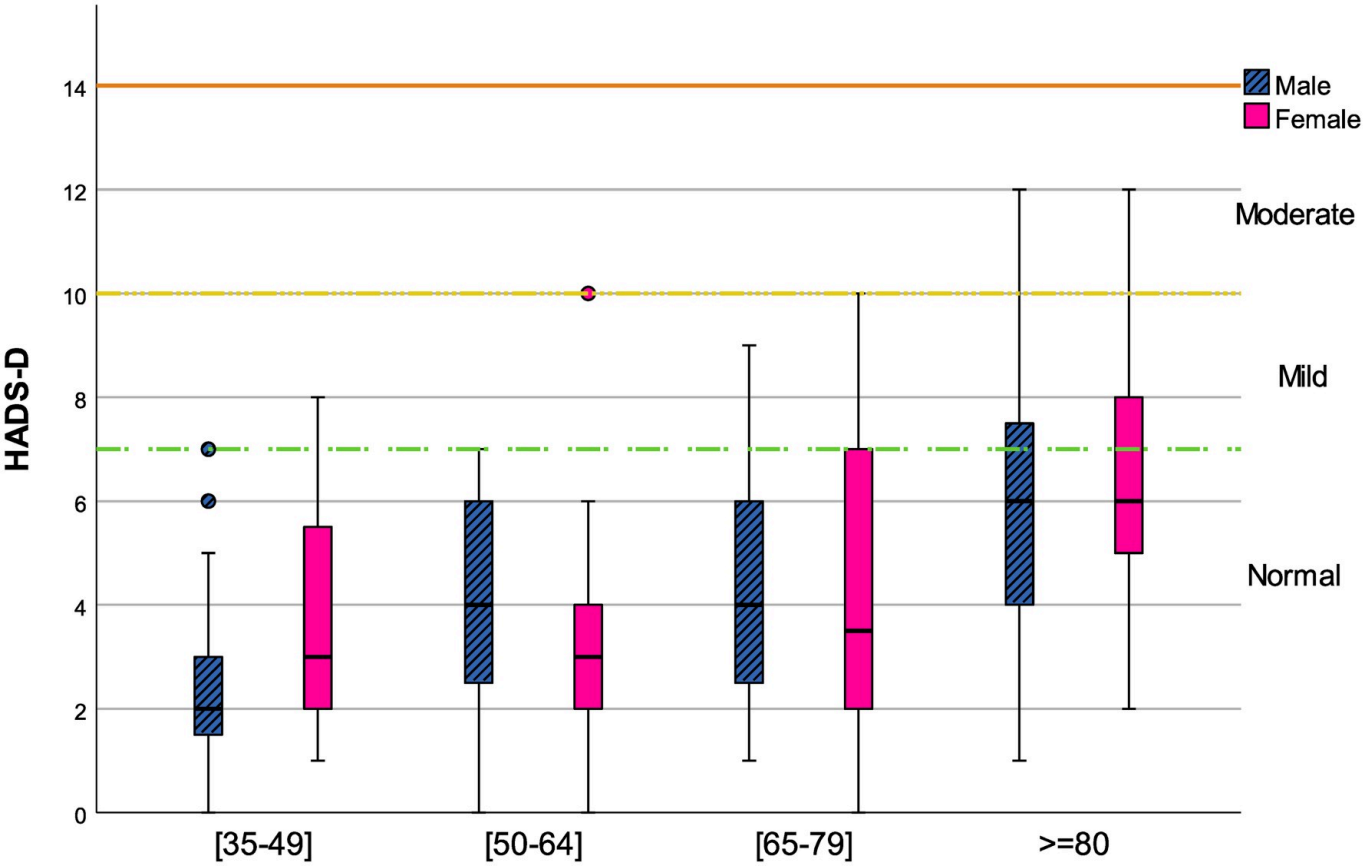
Age effect on HADS-D for both genders.

Table 2 presents the sample characterization concerning demographic variables and mood measures. Concerning age and gender, the sample is almost balanced. Regarding mood measures, for HADS-A and HADS-D, the number of participants with and without presence of anxiety or depression symptoms is unbalanced (26.8% and 10.7%, respectively) and non-severe (HADS-A: 5.4 ± 2.9 and HADS-D: 4.2 ± 2.7, respectively). HADS-A and HADS-D mean score by age group and gender are also presented in Table 2. Figs 1 and 2 represent the age effect on HADS-A and HADS-D, respectively.

**Table 2:** Sample characterization concerning demographic variables and mood measures.

Variables	ALL (n = 112)	Female	Male
Age (years; M ± SD)	62.1 ± 15.6	61.6 ± 15.8	62.6 ± 15.5
Gender (n, %)	112 (100%)	56 (50%)	56 (50%)
HADS_ A >7 (n, %)	30 (26.8%)	20 (35.7%)	10 (17.9)
HADS_A [0-21] (M ± SD)	5.4 ± 2.9	5.9 ± 3.3	4.8 ± 2.3
by age (years): [35-49]	5.8 ± 2.8	6.5 ± 3.2	5.1 ± 2.4
[50-64]	6.0 ± 3.2	6.9 ± 3.6	5.1 ± 2.6
[65-79]	4.8 ± 2.4	5.3 ± 2.9	4.3 ± 1.7
≥80	4.6 ± 2.8	4.4 ± 3.2	4.8 ± 2.5
HADS_ D >7 (n, %)	12 (10.7%)	8 (14.3%)	4 (7.1%)
HADS_D [0-21] (M ± SD)	4.2 ± 2.7	4.3 ± 2.8	4.1 ± 2.6
by age (years): [35-49]	3.2 ± 2.1	3.8 ± 2.2	2.7 ± 2.0
[50-64]	3.8 ± 2.5	3.7 ± 2.9	4.0 ± 2.2
[65-79]	4.2 ± 2.6	4.2 ± 2.9	4.3 ± 2.4
≥80	6.2 ± 2.9	6.4 ± 3.0	6.1 ± 3.0

Concerning HADS-A (Fig 1), there was a tendency for the median values to decrease after the middle age in female participants; in male participants, the age group [65-79] presented the lower median value of HADS-A. Only females of the age groups [35-49] and [50-64] presented part of the boxplot whiskers above the cut-off of 10 (moderate symptoms of anxiety). Although, two-way ANOVA showed no statistical effect of age (*F*(3, 104) = 1.618; *p* = 0.190) or gender (*F*(1, 104) = 3.039; *p* = 0.084) on HADS-A scores. Additionally, significant interaction between age group and gender for HADS-A (*F*(3;104) = 0.692; *p* = 0.559) was not detected.

HADS-D (Fig 2) on male participants increases continuously with age and in female participants there is a sharper increase in older age groups. HADS-D tends to increase with age. In the older age group, for both genders, the boxplot whiskers achieved the moderate symptoms of depression, but all median values are observed in the normal range. The ANOVA results showed a significant effect of age on HADS-D (*F*(3;104) = 6.065; *p* = 0.001), with significant differences between the age group ≥80 and all the younger groups, but no significant statistical effect of gender (*F*(1;104) = 0.275; *p* = 0.601). Additionally, the interaction of age with gender was non-significant (*F*(3;104) = 0.470; *p* = 0.704).

### Association of HADS-A and HADS-D with acoustic parameters

Considering the division of HADS scores in absence ([0-7]) and presence of symptoms (>7), the mean and SD values of all acoustic parameters by gender and mood symptoms are presented in Table 3.

**Table 3:** Acoustic parameters’ characterization by HADS sub-scores and gender.

	Variables	Gender	HADS-A		HADS-D	
			≤7	>7	≤7	>7
			n = 36 (F) + 46 (M)	n = 20 (F) + 10 (M)	n = 48 (F) + 52 (M)	n = 8 (F) + 4 (M)
**Segmental**	**Vowels F0 (Hz)**	F	192.0 ± 25.8	190.5 ± 20.0	192.6 ± 24.3	184.9 ± 20.3
		M	137.4 ± 30.0	153.1 ± 25.8	137.9 ± 26.6	170.3 ± 53.0
	**Vowels F1 (Hz)**	F	499.9 ± 34.9	499.5 ± 24.6	500.8 ± 32.8	493.6 ± 21.4
		M	444.9 ± 26.7	451.0 ± 25.9	446.3 ± 27.4	442.1 ± 6.5
	**Vowels F2 (Hz)**	F	1682.0 ± 62.8	1653.2 ± 56.8	1671.9 ± 63.2	1670.6 ± 56.2
		M	1426.9 ± 59.5	1418.8 ± 62.9	1423.7 ± 57.7	1448.1 ± 88.5
	**Vowel duration (s)**	F	137.2 ± 28.4	124.8 ± 19.5	130.5 ± 23.1	146.2 ± 39.1
		M	127.9 ± 23.1	126.6 ± 24.6	126.5 ± 23.0	142.8 ± 21.9
**Suprasegmental**	**Total speech duration (s)**	F	23.6 ± 14.5	18.5 ± 9.9	22.7 ± 13.9	16.6 ± 5.8
		M	20.8 ± 11.8	20.8 ± 16.7	21.5 ± 12.8	11.5 ± 4.5
	**Total pause duration (s)**	F	7.6 ± 5.4	9.1 ± 7.0	7.7 ± 5.3	10.7 ± 9.2
		M	8.6 ± 5.2	7.8 ± 5.3	8.5 ± 5.2	6.8 ± 5.3
	**Total recording duration (s)**	F	31.2 ± 17.5	27.6 ± 15.2	30.4 ± 17.1	27.3 ± 14.2
		M	29.4 ± 15.3	28.6 ± 20.6	30.1 ± 16.3	18.3 ± 9.6
	**Percent pause time (%)**	F	24.4 ± 12.6	31.1 ± 11.3	25.9 ± 11.2	32.0 ± 18.5
		M	29.6 ± 10.9	28.9 ± 12.7	29.2 ± 11.2	33.0 ± 10.8
	**Speech pause ratio**	F	8.9 ± 25.1	2.7 ± 1.5	6.8 ± 21.6	5.9 ± 9.9
		M	2.8 ± 1.4	3.0 ± 1.5	2.9 ± 1.4	2.3 ± 1.1
	**Number of pauses**	F	8.2 ± 5.8	8.0 ± 4.7	8.2 ± 5.5	8.1 ± 4.9
		M	8.9 ± 5.0	8.9 ± 7.6	9.2 ± 5.5	5.3 ± 3.7
	**Mean pause duration (s)**	F	1.0 ± 0.6	1.2 ± 0.5	1.1 ± 0.6	1.1 ± 0.7
		M	1.0 ± 0.4	1.0 ± 0.5	1.0 ± 0.4	1.3 ± 0.2
	**Mean speech duration (s)**	F	3.1 ± 3.0	2.2 ± 0.7	2.9 ± 2.6	2.1 ± 0.8
		M	2.1 ± 0.7	2.1 ± 0.8	2.1 ± 0.7	2.1 ± 0.5
	**Pause variability (s)**	F	0.6 ± 0.6	0.7 ± 0.6	0.6 ± 0.5	0.9 ± 0.9
		M	0.6 ± 0.5	0.6 ± 0.7	0.6 ± 0.6	0.6 ± 0.5
	**Speech variability (s)**	F	1.5 ± 0.7	1.4 ± 0.6	1.5 ± 0.7	1.1 ± 0.5
		M	1.2 ± 0.6	1.3 ± 0.4	1.2 ± 0.5	1.1 ± 0.3
	**Number of syllables**	F	111.0 ± 76.8	89.4 ± 50.1	107.8 ± 72.4	76.0 ± 31.5
		M	108.9 ± 61.1	98.0 ± 68.5	110.5 ± 62.6	60.3 ± 26.0
	**Speech rate (syllables/s)**	F	3.5 ± 1.0	3.3 ± 0.8	3.5 ± 0.9	3.1 ± 1.0
		M	3.8 ± 0.9	3.6 ± 0.7	3.7 ± 0.9	3.4 ± 0.3
	**Speaking F0 (Hz)**	F	190.5 ± 21.0	189.6 ± 20.6	191.6 ± 20.8	181.8 ± 19.1
		M	123.5 ± 19.6	141.9 ± 26.5	124.9 ± 20.7	151.5 ± 24.9
	**HNR (dB)**	F	14.4 ± 2.2	14.3 ± 2.3	14.4 ± 2.1	14.0 ± 2.9
		M	10.3 ± 2.1	10.8 ± 2.3	10.3 ± 2.1	12.2 ± 1.0

F: female; M: male

Table 3 was analyzed based on the intensity and direction of change of each acoustic parameter, and changes higher than 10% [11] are reported next. Considering HADS-A, in the group of female speakers with anxiety symptoms, an increase occurs in total pause duration, percent pause time and mean pause duration; a decrease arises in total speech duration, speech pause ratio, mean speech duration and number of syllables. In male speakers, none acoustic variable presents a change that differs 10% from the mean in the group of speakers with anxiety symptomatology. Although, speaking F0 and number of syllables were the acoustic variables that present the highest increase (7,5%) and the largest decrease (-5.0%), respectively, in the group of speakers with anxiety symptoms.

Regarding depressive symptoms, for females, an increase is observed in total pause duration, percent pause time and pause variability. A decrease occurs in total speech duration, mean speech duration, speech variability and number of syllables. For males, the acoustic variables that present an increase higher that 10% are vowel F0, mean pause duration and speaking F0. A decrease superior to 10% occurs in the acoustic variables total speech duration, total pause duration, total recording duration, speech pause ratio, number of pauses and number of syllables.

For a more in deepth analysis of the association between the independent variables (i.e., acoustic variables: vowels F0, vowel duration, vowels F2, total speech duration, total pause duration, speech rate, percent pause time and HNR) and the dependent variable (HADS-A or HADS-D scores), a multiple linear regression model was applied and adjusted by the influence of age and gender. In Table 4 the multiple regression model results for HADS-A and HADS-D are presented. Although no significant gender differences were observed for both HADS sub-scales (see Figs 1 and 2), and only HADS-D presented significant age differences, this approach is justified by the influence of these demographic variables on anxiety/depressive symptoms in other studies in this field [75–81]. For HADS-A, none of the acoustic variables considered presented a significant effect in both models.

**Table 4:** Results of multiple regression model for HADS-A and HADS-D.

Variables	HADS-A				HADS-D			
	Multivariable Model		Adjusted Model		Multivariable Model		Adjusted Model	
	Coeff. 98.75% CI	*p-value*	Coeff. 98.75% CI	*p-value*	Coeff. 98.75% CI	*p-value*	Coeff. 98.75% CI	*p-value*
**Constant**	4.765 [-7.504–17.034]	*0.326*	18.903 [-0.597–38.404]	*0.015*	5.108 [-5.834–16.049]	*0.238*	6.896 [-10.483–24.276]	*0.315*
**Vowels F0 (Hz)**	-0.002 [-0.029–0.025]	*0.869*	-0.008 [-0.035–0.019]	*0.438*	0.002 [-0.022–0.025]	*0.871*	0.002 [-0.022–0.026]	*0.853*
**Vowel duration (s)**	-0.021 [-0.049–0.008]	*0.067*	-0.015 [-0.046–0.017]	*0.237*	0.026 [0.00–0.051]	*0.011* *	0.012 [-0.016–0.040]	*0.270*
**Vowels F2 (Hz)**	0.000 [-0.007–0.008]	*0.927*	-0.008 [-0.019–0.004]	*0.092*	-0.001 [-0.008–0.005]	*0.611*	-0.003 [-0.013–0.007]	*0.455*
**Total speech duration (s)**	-0.023 [-0.131–0.086]	*0.596*	-0.023 [-0.131–0.084]	*0.582*	-0.130 [-0.227–-0.033]	*0.001* *	-0.112 [-0.208–-0.016]	*0.004* *
**Total pause duration (s)**	-0.005 [-0.302–0.292]	*0.969*	-0.010 [0.304–0.283]	*0.929*	0.284 [0.019–0.549]	*0.008* *	0.249 [-0.012–0.510]	*0.017*
**Speech rate (syllables/s)**	-0.042 [-1.018–0.934]	*0.913*	-0.126 [-1.126–0.873]	*0.748*	-0.059 [-0.929–0.811]	*0.863*	-0.286 [-1.177–0.605]	*0.417*
**Percent pause time (%)**	0.042 [-0.094–0.178]	*0.437*	0.053 [-0.080–0.187]	*0.314*	-0.086 [-0.208–0.035]	*0.074*	-0.090 [-0.209–0.029]	*0.057*
**HNR (dB)**	0.214 [-0.116–0.544]	*0.103*	0.119 [-0.217–0.455]	*0.371*	0.052 [-0.243–0.347]	*0.655*	0.074 [-0.226–0.373]	*0.533*
**Age**	–	–	-0.028 [-0.080–0.024]	*0.177*	–	–	0.047 [0.001–0.094]	*0.010* *
**Gender (Female)**	–	–	3.093 [-0.297–6.483]	*0.022*	–	–	0.439 [-2.582–3.460]	*0.712*

Coeff.: Unstandardized Coefficient;

* *p*<0.0125

For depression symptoms, expressed by the HADS-D scores, vowel duration, total speech duration and total pause duration presented significant effects. However, in the adjusted model only total speech duration maintain the significant effect, along with age. In the adjusted model, age was also significantly associated with HADS-D.

**Fig 3:**
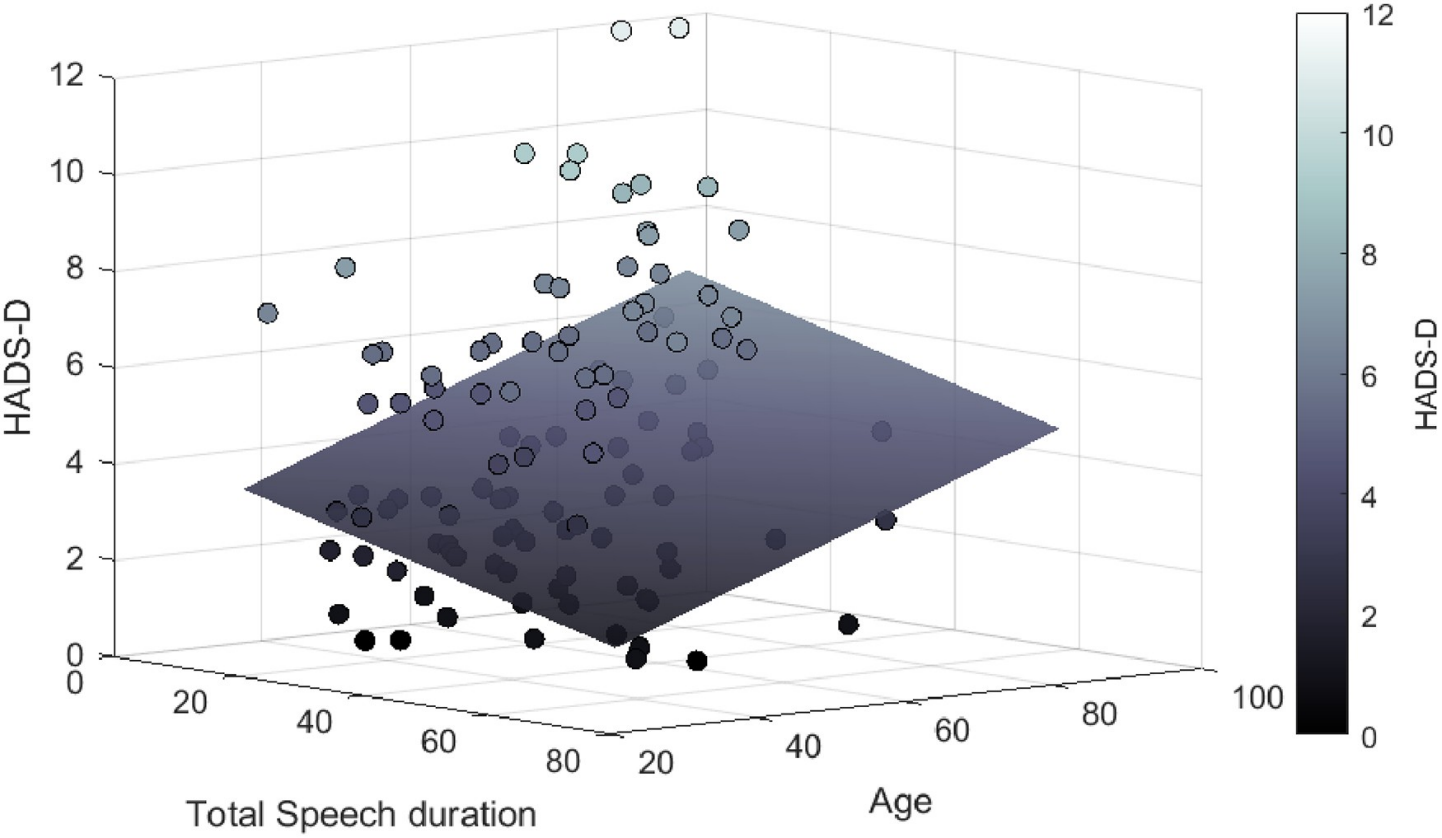
Relationship between total speech duration, age and HADS-D.

Fig 3 demonstrates the association of the depression symptoms scores and the total speech duration and age. The increase of depressive symptoms is related to the total speech duration decrease and to age increase.

## Discussion

The present study aimed to analyze the relationship between the scores of the HADS questionnaire and the segmental acoustic parameters (e.g., F0, F1, F2 and duration of stressed vowels) and also the suprasegmental measures obtained in a sample of 112 individuals (aged 35 to 97) with non-severe mood symptoms. The aim of the study was achieved considering the general alignment of our results with previously reported research related with mood diagnosed disorders.

Regarding anxiety symptoms, there are no acoustic variables that presented a significant association with HADS-A scores. The independent variables used to develop the multiple linear regression do not present a high increase/decrease difference between participants without anxious symptoms and participants with anxious symptoms (see Table 3). In fact, the majority of those differences were below 5%, which can be an explanation for the non-significance observed in the multiple linear regression. The authors can argue that this minor difference could not be sufficient to make the acoustic variables sensitive to sub-clinical anxiety symptoms. Additionally, the observed tendency to higher HADS-A values in younger females has been reported in other studies [76, 79–81], due to interactions between behaviors, internal gender characteristics and stressors [82].

For depression symptomatology, this study presents significant results for both segmental and suprasegmental levels. At segmental level, vowel duration presents a significant effect of the depressive symptoms, meaning that vowel duration increases as depressive symptoms increase. The significant effect of depressive symptoms in vowel duration found in the present study, that analyzed a sample mostly constituted by speakers with non-severe depressive symptoms, also highlight the importance of segment duration in the identification of mood signs. The current results are in line with Trevino et al. [45], that, through the use of phone-duration measures instead of global measures of speech rate, found significant positive correlations between the duration of some vowels with the worsening of depression. To reinforce the results obtained in the present study the findings of Alghowinem et al. [83] and Honig et al. [32] can also be reported, which concluded that syllable duration (in average) were significantly higher in the group of depressed individuals.

At suprasegmental level, first, the total pause duration increases with more depressive symptoms, and the total speech duration present an inverse trend. Mundt et al. [4] and Mundt et al. [34] revealed that great depression symptoms result in more and longer pauses, which was also reflected in a higher total pause time, as occurred in the present research. Conversely, in both studies [4, 34] more time was needed to deliver the message (i.e., more total speech duration). However, other studies have reported that speakers with depressive symptoms exhibit a decrease in speech time or in verbal productivity [84–86]. That is, these speakers tended to produce fewer words [86] and to decrease the phonation time (i.e., utterances are shorter in duration and are less numerous) [84]. Results concerning the increase in total pause duration and the decrease in total speech duration (the one that maintain the significant effect on the adjusted model) could be considered an index of psychomotor retardation or lower cognitive function, and affect the amount of information content to be communicated [84]. In the present study, the total speech duration decreases in speakers with more depressive symptoms (a difference of -23.3% for participants with depressive symptoms), due to the fact that spontaneous speech production is more cognitively demanding in comparison with automatic speech/reading tasks, requiring preparation, word selection and higher motor articulatory control [4, 84]. The increase in total pause duration in participants with more depressive symptoms could suggest more efforts in communication planning and higher cognitive elaboration time [84]. The current results (i.e., significant effect of HADS-D on total pause duration and total speech duration) also highlight the importance of rhythmic measures assessed in spontaneous speech for depression symptoms recognition.

Additionally, although the acoustic variable number of syllables has not entered in the regression model, due to the high correlation with total speech duration, in the descriptive data a decrease of more than 10% in the number of syllables was observed for both genders with score >7 on HADS-D (see Table 3). The number of syllables decrease may be related with the total speech duration decrease and the total pause duration increase with the depression worsening.

Although speech rate in spontaneous speech does not present a significant effect in depressive symptoms, considering that vowels constitute the syllable nucleus, an increase in the time needed to produce a vowel could contribute to a decrease in syllable production per time unit [7] and, consequently, a decrease in speech rate in reading task. Speech rate is referred in the literature as one of the most strongly associated acoustic features with depression status [5] and also one of the first symptoms of depressive disorders, observable by interlocutors [87]. The literature indicates that individuals with more depressive symptoms present lower speech rates [25, 30, 34, 38, 88], even in brief sadness induction [89]. The sensitivity of speech rate for recovery of depressive symptoms has also been evidenced, as the improvement in symptomatology has a positive influence on speech rate [27, 33].

The significant findings mentioned above concerning the rhythmic measures (i.e., total pause duration) and vowel duration do not maintain the effects on the adjusted model by age and gender, which provides evidence of a greater influence of age in mood symptoms. Age is the demographic variable that presents a significant effect on the depressive symptoms assessed by HADS-D. Depression symptoms presented statistically significant higher values in older adults, which is in accordance with studies developed in low-income countries [75, 78]. On epidemiologic studies in Western countries the rate of depression decreases with age, which is the opposite performance of depression mean values across age in the present study. Balabanova and MacKee [90] and Bobak et al. [91] suggest that high numbers of depression symptoms in older ages could mirror high levels of poverty or poor physical health. Bromet et al. [75] also suggest that an increase of depression in elderly could reflect negative changes in social support and in subjective health. The largest European research study on aging (DO-HEALTH) [92] concludes that elderly individuals in Portugal present low levels of cognitive and physical health compared to other European countries. In fact, only 9% of the Portuguese sample are considered healthy, much lower than the 58% from Austria, 51% from Switzerland or 38% from Germany. Portuguese researchers from the DO-HEALTH study revealed that different social resources could explain poor health levels, including level of education, values of pensions or ease access to health care.

Several differences found between the present study and previous research could be due the presence to having participants with absent-to-mild symptoms whereas most studies also include individuals with severe mood symptoms.

### Study limitations

This study presented some limitations. The first limitation is related with the task nature used to extract suprasegmental features once the older speakers performed smaller descriptions than younger adults. This may be related with task or indicate differences in linguistic domain [93]. Lastly, some results should be considered with caution due to the recording environment and the automatic extraction procedures, considering that labelled syllables were not manually verified, but they were obtained in a standardized way for all speakers. Additionally, while certain acoustic features were found to be associated and important in explaining depression and anxiety symptoms, machine learning models would be needed to determine how important they are predicting these mental health states.

## Conclusion

The results of this study lead to different conclusions, concerning the impact of anxiety/depression symptoms on acoustic features extracted by a self-assessment of mood in a sample of adult individuals aged 35-97.

For the individuals of the present study, mainly constituted by adults with non-severe mood symptoms, an increase in depressive symptoms is associated with higher vowel duration, increase of total pause duration and less total speech duration in the univariable model. Adjusting the linear model for age and gender revealed that age affects the depressive symptoms. Only the total speech duration decrease in the adjusted model, along with age, maintain the significant relationship with depression symptoms. Contrariwise, an increase of the anxiety symptoms did not present significant relationships associated with the acoustic parameters studied.

The present study reports the association between non-severe symptoms of anxiety/depression and segmental and suprasegmental acoustic features, constituting an advance in this research field. However, and considering the study limitations, future research studies intend to analyze acoustic features extracted from other speech samples (e.g., text reading) in a group of individuals with a diagnose of anxiety and/or depression compared with a control group across lifetime.

## Supporting information

S1 File Database. Speakers information concerning HADS scores, acoustic measures and demographic variables.
(XLSX)
